# Physical Health and Work Ability among Healthcare Workers. A Cross-Sectional Study

**DOI:** 10.3390/nursrep12020026

**Published:** 2022-04-05

**Authors:** Giacomo Garzaro, Marco Clari, Catalina Ciocan, Beatrice Albanesi, Gloria Guidetti, Valerio Dimonte, Ilaria Sottimano

**Affiliations:** 1 Department of Public Health and Pediatrics, University of Torino, 10126 Turin, Italy; giacomo.garzaro@unito.it (G.G.); catalina.ciocan@unito.it (C.C.); beatrice.albanesi@unito.it (B.A.); valerio.dimonte@unito.it (V.D.); 2 Città della Salute e della Scienza University Hospital, 10126 Turin, Italy; 3 Dipartimento di Scienze Psicologiche, della Salute e del Territorio University G. D'Annunzio, 66100 Chieti-Pescara, Italy; gloria.guidetti@unich.it; 4 Department of Psychology, University of Torino, 10124 Turin, Italy; ilaria.sottimano@unito.it

**Keywords:** aging, health personnel, nurses, physicians, work ability

## Abstract

Healthcare workers’ age is increasingly rising, negatively affecting their physical health. In particular, workability is an emerging phenomenon that predominantly affects healthcare workers. This study aims to assess physical health status and workability among ageing healthcare workers. A cross-sectional study using the Work Ability Index (WAI) was performed. Data were collected in a university hospital in northern Italy. Data were collected voluntary through a questionnaire. Healthcare workers participating in the survey were contacted personally by two resident physicians. Thus, the total number of study participants was 220 among nursing aides, nurses, and physicians. Data were analyzed by performing ANOVA and regression to assess the differences between the healthcare workers and age groups. A generalized linear model was tested to evaluate the effect of age and task on workability. The majority of healthcare workers had good WAI values. Physicians’ workability was higher than nursing aides. Nursing aides suffered more from cardiovascular disorders, while physicians and nurses had more musculoskeletal disorders. However, the distribution was statistically different (χ^2^ = 24.03, *p* = 0.00), as most of the physicians’ workability values were good and good, while those of nursing aides and nurses were good and medium. In line with previous studies, the decrease in WAI with ageing is strictly dependent on the type of task assigned. Due to heavy physical tasks, nurses and nurses’ aides showed a greater WAI than physicians. This study highlights the critical issues faced by ageing healthcare professionals. In the near future, it is necessary to find solutions to cope with these changes and devise possible interventions aimed at ameliorating workability.

## 1. Introduction

In the last few years in all Western countries, the age of the working population has gradually increased. The move from a relatively young to a relatively old workforce is a phenomenon known as “workforce aging”. In particular, the number of workers aged 55 or over is expected to increase significantly over the next few decades, especially in some European countries such as Spain, Italy, Portugal, Greece, and Ireland [1].

As reported by numerous studies, aging leads to several changes that can be either positive or negative. While some aspects, such as wisdom, expertise, strategic thinking, and judgment, generally improve with age, functional abilities, such as those concerning the physical (e.g., muscle strength and bone, aerobic, and cardiac functions) and sensory domains (e.g., sight and hearing), tend to decrease [2,3]. 

Aging also leads to greater vulnerability to hazards with increased risk for work accidents [4] and to increased difficulty in managing psychophysical overload due to worsening health conditions [3,4]. In this regard, Harman argued that aging has become the most important disease risk factor in developed countries [5]. This standpoint is supported by the fact that, as the population ages, there is a substantial increase in chronic illnesses, such as oncological and cardiovascular diseases, as well as musculoskeletal, metabolic, and mental disorders, all becoming more frequent after the age of 55 [6,7].

According to a prominent European survey, in 2011, more than 30% of workers aged between 50 and 64 years had at least one functional limitation in their lower or upper limbs or displayed impaired fine movements as well as being affected by two or more chronic diseases [8]. These aspects are inevitably associated with a decreased work ability [9,10]. This work-related domain was first developed in the late 1980s by the Finnish Institute of Occupational Health (FIOH) to assess the workers’ response to job demands [11] and identify those workers at risk of imbalance between work demands and personal resources. Indeed, one of the main determinants of impaired work ability is age [12], probably because resources tend to change and diminish with aging, while job demands remain quite stable over time [13]. In recent years, many studies have shown that workability is negatively affected by not only age but also high levels of physical and psychosocial work demand [13,14,15], unhealthy lifestyles, and poor physical fitness [16,17,18,19]. In turn, low job skill levels increase sick leave, early retirement, and intention to leave the job [19]. Furthermore, diminished work ability correlates with higher levels of work stress, depression [14,17], and emotional exhaustion [20]. By contrast, good workmanship is often associated with high productivity and predictive of a better quality of life [14].

These issues are particularly relevant to the healthcare system, where interpersonal responsibility is closely intertwined with the various occupational risks to which healthcare providers are routinely exposed, especially for the older workers. Only in 2019, 36% of healthcare workers in EU were aged 50 or over; particularly, in Italy, 45% of people in health occupations were older workers [20].

Several reports have shown how shift work, especially night work, fairly common among healthcare professionals, can negatively affect the workers’ psycho–physical balance, performance efficiency and extra-work relationships—shift workers are in fact more prone to human errors and accidents due to altered circadian rhythms [21]. Furthermore, shift work is an important health risk factor for cardiovascular, gastrointestinal, psychological, and neoplastic diseases [21,22,23]. These aspects become even more relevant with aging, as it is much more difficult for elderly workers to restore their psychophysical balance undermined by night shifts. This also leads to a greater incidence and severity of sleep disorders, reduced tolerance for prolonged working hours, and decreased work ability, which appears to be more pronounced among shift workers [23]. To make matters worse, reduced physical capacity affects the ability of healthcare professionals to properly handle their patients [23], an emerging issue that has been associated with increased rates of employee’s limitations and unfitness to work [9]. Lastly, adequate patient-healthcare professional relationships require emotional stability and the availability of appropriate relational resources, which are usually associated with increased age and experience [24]. However, these social and behavioral skills are quite often disrupted by anxiety and mood disorders, which also tend to increase with aging [25]. 

Understanding the workability of healthcare professionals appears to be particularly important in light of the current situation of healthcare systems, plagued by hiring freeze and prolonged working life due to the economic crisis. Thus, this study aims to assess the physical health status and workability among aging nursing aides, nurses, and physicians.

## 2. Materials and Methods

### 2.1. Procedures

A cross-sectional study was conducted collecting data in 2018. Health workers participating in the survey were contacted personally by two resident physicians. In accordance with current privacy legislation, all the participants were informed of the aims of the research project and were told that the data obtained would be used only for research purposes and processed in an anonymous and aggregate fashion. After this disclosure, the participants were asked to answer a questionnaire on a voluntary basis. In the same disclosure, it was also stated that the response to the questionnaire would have been regarded as expressed consent to treat personal data. The completion of the questionnaire as well as the reading and signing of the informed consent occurred during working hours at a university hospital in northern Italy.

The study reporting was also consistent with the “Strengthening the Reporting of Observational studies in Epidemiology” (STROBE) checklist (Supplementary File S1). The research followed all the institutional and governmental rules for the ethical use of healthy human volunteers.

### 2.2. Tools

The instruments were chosen and used based on the emerging issue of work sustainability among aging workers, with particular attention to the health context. Workers were thus administered a questionnaire consisting of two sections. The first section investigated some socio-demographic factors (e.g., gender, age, marital status, and children), employment status (e.g., type of contract, working hours, and length of service), and some personal variables (e.g., presence of elderly family members requiring care and availability of facilities provided for by Law 104/92). The second section focused on the workability, measured through the Italian version of the Work Ability Index (WAI) [26]. In the study sample, the internal consistency was found to be satisfactory (Cronbach’s α = 0.72).

The WAI is composed of seven subscales: (1) currently perceived workability in comparison with the best period of life (1 item); (2) workability perceived in relation to the task demands (2 items); (3) number of pathologies declared, supported by relative diagnoses, at the time of completing the questionnaire; (4) subjective estimate of the workability reduction due to the pathologies declared—the disease inventory consisted of 51 pathologies; (5) sick leaves in the last 12 months (1 item); (6) workability estimate for the upcoming two years (1 item); and (7) perception of personal resources in relation to the functional activities performed daily by the individual (3 items). The sum of the scores obtained at seven subscales defines a total score (workability index) that can range from 7 to 49. The WAI score can also be traced to 4 macro-categories: poor (range: 7–27), medium (28–36), good (37–43), and very good (44–49).

### 2.3. Data Analysis

Data analysis was carried out by SPSS 25 statistical software Armonk, NY, USA: IBM Corp. [27]. In order to estimate the prevalence of the phenomena under examination, for each indicator, we calculated the percentages of subjects with at least one diagnosis or falling within the categories defined by the workability cut-off value.

The chi-square test was used to identify any differences in the distribution of physical health disorders with respect to age groups and tasks. With regard to the workability, one-way ANOVA analyses were also performed to assess the presence of any significant differences between the different healthcare workers and age groups. In order to evaluate the effect of age and task on the workability, a general linear model was tested to evaluate the factorial ANOVA.

## 3. Results

### 3.1. Study Participants

The hospital staff participating in the survey consisted of 238 workers performing four tasks: nursing aides (*n* = 73, 30.7%), nurses (*n* = 73, 30.7%), physicians (*n* = 74, 31.1%), and technical-administrative staff (*n* = 18, 7.6%). Because of the specific interests of the survey, which mainly focused on healthcare professionals, and given the low number of non-medical participants, all technical-administrative personnel were excluded. Thus, the total number of study participants was 220, which consisted of three categories: (1) nursing aides (33.3%), (2) nurses (33.3%), and (3) physicians (33.6%). Table 1 shows the socio-demographic characteristics of the study sample.

Among the socio-demographic characteristics of the sample, the nursing aides and nurse groups mainly comprised women albeit women were more frequently present in the nurse group compared to the nursing aides group (χ^2^ = 10.06, *p* = 0.02). The distributions by age group and type of contract—predominantly full-time for both professional categories—were similar. Furthermore, both groups had comparable distributions by marital status: more than 50% of these healthcare professionals were married and had at least one child. Lastly, about 40% of nursing aides and nurses also had other family members to care for, and about 15% of them availed themselves of the benefits provided under Law 104/92.

When physicians are taken into account, a few differences emerged. Physicians were predominantly men, young, and single. In particular, there was a substantial difference in distribution by gender (χ^2^ = 17.37, *p* = 0.00), age (2 = 47.34, *p* = 0.00), and marital status (χ^2^ = 20.64, *p* = 0.00). Furthermore, all physicians had full-time contracts and no family members to care for (children: χ^2^ = 20.69, *p* = 0.00; adults: χ^2^ = 8.77, *p* = 0.01; 104/92: χ^2^ = 9.46, *p* = 0.01). The age differed significantly among the three groups (F = 15.66; *p* = 0.00). Specifically, the nursing aides were the oldest workers with a mean age of 48 years, whereas the nurses were, on average, 46 years old. Interestingly, the physicians were the youngest of all three groups, with a mean age of 39 years. Lastly, the body mass index was normal in most of the sample.

### 3.2. Physical Health Status and Workability

The physical health status was assessed by the WAI item 3, which recorded the presence of 13 physical disorders diagnosed by a doctor (e.g., cardiovascular, musculoskeletal, mental disorders, etc.). In all three healthcare worker groups, it was observed a similar distribution of the following pathologies: respiratory, mental, gastro-intestinal, and genitourinary disorders; oncological diseases; nephropathies; and birth defects (Table 2). In contrast, different distribution patterns emerged with regard to the following pathologies: cardiovascular disorders, more frequent among nursing aides (χ^2^ = 11.28, *p* = 0.02); musculoskeletal disorders, significantly more present in the nurse group (χ^2^ = 24.57, *p* = 0.00); neurological-sensory disorders, more frequently observed among nurses and physicians (χ^2^ = 14.93, *p* = 0.00); and metabolic/endocrinological disorders, mainly affecting nursing aides (χ^2^ = 15.97, *p* = 0.00).

Among nursing aides, cardiovascular disorders had the highest incidence (28.8%), while musculoskeletal disorders had the greatest prevalence among nurses and physicians (39.7% and 21%, respectively) (Table 2).

Overall, the majority of workers had good WAI index values: 38.4% of nursing aides, 41.2% of nurses, and 60.6% of physicians. The other workers were grouped in the more extreme categories: 16.4% of nursing aides, 13.2% of nurses, and 25.4% of physicians reported very good workability, while 7% of nursing aides, 3% of nurses, and no physicians reported poor workability (Table 3). However, the distribution was statistically different (χ^2^ = 24.03, *p* = 0.00), as most of the physicians’ workability values were between good and very good, while those of nursing aides and nurses were between good and medium. This observation also holds true when WAI average scores were considered, which were considerably higher among physicians compared to both nurses and nursing aides (F = 12.78, *p* = 0.00).

Taking into account physical disorders and WAI, nursing aides with cardiovascular disorders had a significantly lower WAI compared to that of nursing aides with good workability (t = 5.23, *p* = 0.00). Similarly, nurses suffering from musculoskeletal disorders had a significantly lower WAI than that of nurses with good workability (t = 5.76, *p* = 0.00). Likewise, physicians with musculoskeletal disorder displayed significantly lower WAI than that of physicians with good workability (t = 3.31, *p* = 0.00) (Table 3).

### 3.3. Relationship between Age and Physical Health Status

**Figure 1:**
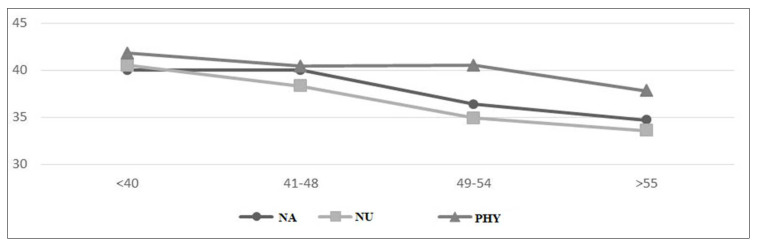
Age distribution of workability. NA, nursing aides; NU, nurses; PHY, physicians.

**Figure 2:**
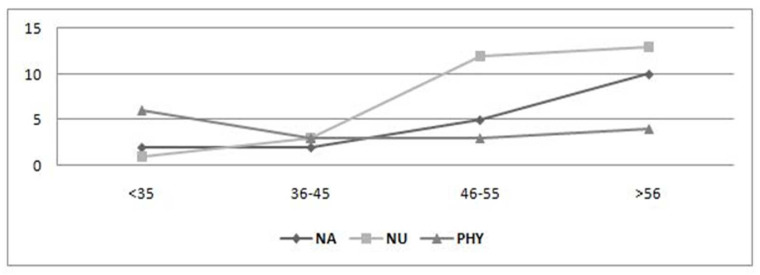
Age distribution of musculoskeletal disorders. NA, nursing aides; NU, nurses; PHY, physicians.

**Figure 3:**
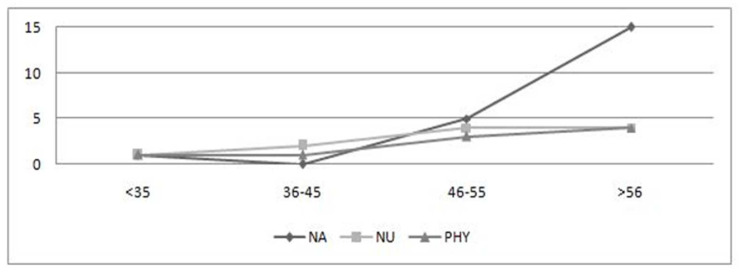
Age distribution of cardiovascular disorders. NA, nursing aides; NU, nurses; PHY, physicians.

Figure 1, Figure 2 and Figure 3 depict the scores of the dimensions investigated among nursing aides, nurses, and physicians in relation to the various age groups. As age increases, physical health conditions worsen in all categories. In particular, the age groups mostly affected were 46–55 and >56 years, especially when it comes to musculoskeletal (Figure 2) and cardiovascular disorders (Figure 3). More specifically, there can be noticed a significant difference in frequency distribution among the age groups with regard to cardiovascular disorders, which were more frequently found in workers aged >56 years, especially for nursing aides (χ^2^ = 20.06, *p* = 0.00) and physicians (χ^2^ = 22.57, *p* = 0.00); musculoskeletal disorders tend to grow with aging among nursing aides (χ^2^ = 19.04, *p* = 0.00) and nurses (χ^2^ = 25.08, *p* = 0.00). The working ability shows a deterioration with the passage of age, in line with the literature (Figure 1). The analysis of variance shows a statistically significant difference among age groups for all worker categories: nursing aides (F = 3.36, *p* = 0.02), nurses (F = 4.15, *p* = 0.01), and physicians (F = 3.92, *p* = 0.01). Considering the combined effect of age and task on the workability, the factorial ANOVA highlighted the presence of a significant main effect of age (F = 8.67, *p* = 0.00) and of the worker’s role (F = 5.71, *p* = 0.00) but not a significant interaction effect (F = 0.62, *p* = 0.71).

## 4. Discussion

The aim of this study was to analyze the physical health status and workability of healthcare workers from the aging perspective. Previous reports have shown higher rates of low workability among nursing aides and nurses compared to physicians [23]. According to these studies, the decrease in WAI with aging is strictly dependent on the type of task assigned; it is greater and starts earlier in workers performing heavy physical tasks (e.g., nurses), whereas it is much less pronounced and delayed in workers carrying out lighter physical activities (e.g., physicians) and on the other white collars [11]. Fittingly, nursing aides and nurses are more likely to display decreased workability at an older age [23], having to deal directly with the basic needs of hospitalized patients, such as mobilization. On the other hand, the WAI remains substantially unchanged for workers mainly involved in intellectual activities.

Impaired workability among nursing aides and nurses is not just the result of high levels of physical work demand, but it is also caused by the intense relational demands inherent to their task of caring for the patients. Indeed, patient–healthcare worker relationships are sometimes characterized by disproportionate patient expectations (i.e., client-related stressors), often leading to verbal aggression [28,29,30]. In addition, workability is also affected by prolonged duration of working days [30] and poor control over the tasks to be performed [31,32]. 

Nursing is generally regarded as a very demanding job [32,33,34] because it requires the use of physical and psychological resources that tend to diminish over time. This inevitably has a negative impact on the workers’ capability of performing tasks, thus lowering their workability [9]. Moreover, this tendency has a negative influence on the “motivation to work” domain, which has been shown to be increasingly entwined with mental and physical health status, stress management, and work organization [30].

By contrast, physicians seem maintain a good workability, which can in part be ascribed to the lower physical demand of their tasks [23] and/or to the fact that these workers are less exposed to prolonged direct contact with patients on a routine basis [35]. Another possible reason for this phenomenon may be related to a higher job satisfaction and a greater control over their work tasks, two determinants that are generally found more frequently among physicians than other healthcare professionals. This explanation would also be in line with previous studies showing a positive correlation between workability and job satisfaction [32] and the protective effect of organizational variables on workability [36].

With regard to physical health disorders, our findings confirm how aging increases the prevalence of chronic diseases, particularly musculoskeletal and cardiovascular disorders, among older workers, especially after the age of 55 [6,7]. The impairment of the physical domain inevitably leads to increased number of workers with low workability [9,10]. Of note, the risk of having cardiovascular disease is much greater in workers with a low level of education, such as nursing aides [37,38]. This implies that level of education is an important predictor of cardiovascular disease [38].

Our findings confirming previous studies on workability are particularly useful with regard to the worldwide healthcare context where the age of professionals has gradually increased due to the delayed retirement and hiring-freeze policies. Therefore, to design and implement health promotion and sustainability interventions at work, it is essential to identify the main risk factors exposed to healthcare workers due to the ageing process.

This study has some limitations: the physical health disorders identified are limited by the generic nature of the information collected, which does not allow us to identify specific musculoskeletal or cardiovascular disorders or assess their severity; in addition, the single-center nature of the research may limit the generalizability of our findings.

## 5. Conclusions

This study highlights the critical issues faced by aging healthcare professionals, especially nursing aides and nurses, performing fundamental tasks in the hospital setting. Workability is a wide area of study, which is increasing in recent years due to the aging progression of workers. In the near future, due to the economic crisis, it is highly likely that the healthcare system will undergo massive workforce reduction paralleled by the increasing number of workers aged over 55 years. The emerged perspective promote an innovative contribution regarding physical health and musculoskeletal conditions. It is therefore necessary to find solutions on how to cope with these changes and to devise possible interventions aimed at making work accessible even for elderly workers. In this regard, previous studies have shown how the implementation of flexible working arrangements, part-time jobs, job sharing, and ergonomic solutions can improve both personal and team effectiveness in the workplace [39,40]. Moreover, high workability in the health sector means lower costs and better service to users, with repercussions on large parts of the population. These solutions, together with a complete assessment of the risk of biomechanical overload [39,40,41], could help make job tasks more viable for aging healthcare workers, especially nursing aides and nurses. 

### Applying Research to Occupational Health Practice

Healthcare workers’ age is increasingly rising, negatively affecting their work ability. Impaired work ability among nursing aides and nurses is not just the result of high levels of physical work demand, but it is also caused by the intense relational demands inherent to their task of caring for the patients. Occupational health nurses working with healthcare professionals need to find solutions on how to cope with the workforce aging and to devise possible interventions aimed at making work accessible even for elderly workers. These interventions should include the promotion of flexible working arrangements, part-time jobs, job sharing, and ergonomic solutions that can improve both personal and team effectiveness in the workplace. More studies about the associated causes of the development of physical conditions are warranted to better investigate the effectiveness of interventions. Further understanding is also needed of the direct costs associated with staff working continuity and the direct costs associated with professionals’ health problems, absenteeism, injuries, and difficulties.

## Figures and Tables

**Table 1:** Socio-demographic characteristics among study participants.

Variable	Nursing Aides *n* (%)	Nurses *n* (%)	Physicians *n* (%)
Sex			
Male	33 (45.2)	15(20.5)	39(52.7)
Female	40 (54.8)	58(79.5)	35(47.3)
Age			
<35	8 (11)	8 (11)	38 (51.4)
36–45	18 (24.7)	25 (34.2)	10 (13.5)
46–55	22 (30.1)	24 (32.9)	14 (18.9)
>56	25 (34.2)	16 (21.9)	12 (16.2)
Work contract			
Full Time	64 (87.7)	66 (94.3)	74 (100)
Part Time	7 (9.6)	4 (5.7)	-
Marital status			
Single	17 (23.3)	20 (27.4)	36 (48.6)
Married/partner	39 (54.4)	42 (57.5)	33 (44.6)
Separated/divorced	10 (13.7)	10 (13.7)	4 (5.4)
Widow/widower	7 (9.6)	1 (1.4)	1 (1.4)
Children			
Yes	55 (75.3)	51 (70.8)	31 (41.9)
No	18 (24.7)	21 (29.2)	43 (58.1)
Other people to care for			
Yes	30 (41.7)	27 (37.5)	14 (19.7)
No	42 (58.3)	45 (62.5)	57 (80.3)
Benefits according to Law 104/92			
Yes	17 (23.3)	10 (14.1)	4 (5.5)
No	56 (76.7)	61 (85.9)	69 (94.5)
Body Mass Index (BMI)			
Underweight	-	2 (2.8)	3 (4.2)
Normal weight	36 (52.2)	48 (67.6)	53 (74.6)
Overweight	27 (39.1)	14 (19.7)	13 (18.3)
Obesity	6 (8.7)	7 (9.9)	2 (2.8)

**Table 2:** Diseases and physical disorders reported within the WAI: frequencies (*n*) and percentages (%).

Diagnosis	Nursing Aides *n* (%)	Nurses *n* (%)	Physicians *n* (%)
Cardiovascular disease	21 (28.8)	11 (15.1)	9 (12.2)
Musculoskeletal disorders	19 (26)	29 (39.7)	16 (21.6)
Respiratory disorders	6 (8.2)	6 (8.2)	3 (4.1)
Mental disorders	3 (4.1)	8 (11)	6 (8.1)
Sensory neurological disorders	6 (8.2)	11 (15.1)	10 (13.5)
Gastrointestinal disorders	11 (15.1)	11 (15.1)	6 (8.1)
Genitourinary disorders	8 (11)	4 (5.5)	3 (4.1)
Dermatological disorders	17 (23.3)	19 (26.0)	8 (10.8)
Oncological disorders	1 (1.4)	-	1 (1.4)
Metabolic and/or endocrine disorders	17 (23.3)	7 (9.6)	2 (2.7)
Nephropathies	-	2 (2.7)	-
Birth defects	-	1 (1.4)	2 (2.7)

**Table 3:** Workability among healthcare professionals.

Variable	Nursing Aides M (SD)	Nurses M (SD)	Physicians M (SD)
Average WAI score	37.1 (6.4)	36.4 (6.1)	40.8 (3.6)
Workability	*n* (%)	*n* (%)	*n* (%)
Very good (WAI 49–44)	12 (16.4)	9 (13.2)	18 (25.4)
Good (WAI 43–37)	28 (38.4)	28 (41.2)	43 (60.6)
Medium (WAI 36–28)	26 (35.6)	28 (41.2)	10 (14.1)
Poor (WAI 7–27)	7 (9.6)	3 (4.4)	-
Workability according to age	M (SD)	M (SD)	M (SD)
<35 years	40.0 (5.5)	40.5 (3.2)	47.8 (3.2)
36–45 years	40.0 (7.0)	38.3 (6.8)	40.4 (3.2)
46–55 years	36.4 (5.8)	34.9 (5.3)	40.5 (4.1)
>56 years	34.7 (5.6)	33.6 (4.9)	37.8 (3.1)

WAI, Work Ability Index.

## Data Availability

The data are not available to the public without the consent of the PI (G.G.).

## Supplementary Materials

The following supporting information can be downloaded at: https://www.mdpi.com/article/10.3390/nursrep12020026/s1, Supplementary File S1: STROBE Statement—Checklist of items that should be included in reports of cross-sectional studies.
